# Knowledge, Attitude and Experiences of Students Regarding Menstrual Cup Usage in a Medical College in North Kerala, India

**DOI:** 10.7759/cureus.46151

**Published:** 2023-09-28

**Authors:** Anupama Arumadi, Rupesh Raman, Nourin A Thayyil, Ruby R Rasheed

**Affiliations:** 1 Community Medicine, Kunhitharuvai Memorial Charitable Trust (KMCT) Medical College, Kozhikode, IND

**Keywords:** kerala, medical students, menstrual hygiene, menstruation, menstrual cup

## Abstract

Background: Sanitary napkins form a major source of solid waste, the disposal of which often results in environmental pollution. Menstrual cups are an eco-friendly and cost-effective alternative to sanitary pads that have yet to gain much popularity among Indian women. The current study aims to assess the knowledge, attitude, and experiences of using the menstrual cup among medical students in a college in north Kerala, India.

Methods: This cross-sectional study was conducted among 109 female medical students. Data was collected through an online platform and analyzed using Epi Info version 7.2 (Centers for Disease Control and Prevention, Atlanta, GA).

Results: Though all the participants had heard about the menstrual cup before, only 14 (12.8%) were using it either alone or in conjunction with sanitary pads. The majority of the participants had a positive attitude towards the usage of menstrual cups with 91.74% considering it a better alternative to other sanitary products, Among the several concerns expressed by the participants, 58.7% were concerned about the insertion of a foreign material into their bodies.

Conclusions: Though most of the participants displayed a positive attitude towards the menstrual cup, the number of participants who had ever tried it was very low. There are several apprehensions regarding the cup that need to be addressed before it can be expected to gain popularity.

## Introduction

Menstrual hygiene is an important aspect of women's health. Poor hygiene and usage of poor-quality menstrual products during menstruation can result in reproductive and urinary tract infections and may have far-reaching consequences [1-3]. Menstrual hygiene materials are the products used to contain the menstrual flow, such as pads, cloths, tampons, or menstrual cups [4]. The choice of menstrual hygiene material depends on sociocultural and economic backgrounds as well as personal comfort. No single material will be preferable to all people who menstruate at all times, and each method will have its own merits and demerits according to the user.

Sanitary napkins are among the most preferred menstrual products in India due to their ease of usage, availability, and diversity of the products [5]. However, they also form a major source of solid waste approximately constituting an annual menstrual waste generation of 113 thousand tonnes in India [6]. According to the Solid Waste Management Guidelines 2016, sanitary napkins must be wrapped securely in pouches provided by the manufacturers or in a suitable wrapping material and placed in the bin designated for dry or non-biodegradable waste. This waste is to be then collected and sent to a solid waste processing facility [7]. However, this requires awareness and segregation at the generator level and large-scale organization and provision of infrastructure by the local authorities for proper disposal. Unsafe practices like throwing, burning, burying, flushing down the toilet, etc. are still prevalent which can have a serious impact on the environment [8,9].

The menstrual cup is a good alternative to disposable menstrual products as it is reusable and environment-friendly. The menstrual cup is inserted into the vagina where it acts as a receptacle for the blood, capable of holding up to 10-38 ml of blood. They are made of medical-grade silicon, rubber, latex, or elastomer and can be used for up to 10 years [10].

Though the first menstrual cup was patented as early as 1937, it has not yet gained much popularity in India [5, 11-13]. Poor knowledge, concerns about leakage, perceived discomfort, poor availability, and lack of promotion are some of the reasons for the decreased popularity of menstrual cups [12-14]. These barriers to the usage of menstrual cups exist even among the educated population despite its several advantages. Hence, the present study aims to assess the knowledge and attitude among medical students regarding the usage of menstrual cups and to explore the experiences of the participants who have used menstrual cups. The study will shed light on the lacunae in knowledge and address the concerns associated with the usage of menstrual cups.

## Materials and methods

This cross-sectional study was conducted in Kunhitharuvai Memorial Charitable Trust (KMCT) Medical College in Kozhikode, Kerala among female medical students during the year 2021.

The nature and objectives of the study were explained to the participants and data was collected through a self-administered online questionnaire. The link was personally sent to the participants through an online platform. The questionnaire was divided into sections and data on the knowledge of the participants about menstrual cups, their attitude towards cups, and the experiences of the participants who were using cups were collected. A total of 109 participants responded to the questionnaire.

Statistical analysis

Data from the online platform was cleaned and entered into an Excel sheet. Statistical analysis was done using Epi Info version 7.2 (Centers for Disease Control and Prevention, Atlanta, GA). Qualitative data are described in terms of frequencies and percentages and quantitative data in terms of means and standard deviations.

Ethical considerations

The study was approved by the Institutional Ethics Committee, KMCT Medical College, Approval No.KMCT/MC/IRC/17911082023. Consent was taken online from the participants before they began the questionnaire and confidentiality of the data was maintained throughout the study.

## Results

A total of 109 students participated in the study. The mean age of the study population was 21.6 ± 1.22 years. Though all the participants had heard about the menstrual cup before, only 14 (12.8%) were using it either alone or in conjunction with sanitary pads. In total, 102 (93.6%) were using sanitary pads on at least one of the days of their period and 88 (80.7%) were using only sanitary pads. Also, all the participants knew that menstrual cups could be reused.

Knowledge and attitude of the participants

Around 9.2% (n=10) of the participants knew that a menstrual cup could be made of silicone, rubber, or latex. However, medical-grade silicon is the most commonly used material, which was the response marked by the majority (59.6%) of the participants; 12 (11%) did not know about the material used. Regarding the frequency of clearing the menstrual cup, 74.3% of the participants knew that the cup should be emptied every 4-12 hours; 13 (11%) responded that they were unaware.

Regarding knowledge of the method of sterilization of the cup, 75 (68.8%) participants knew that the cup should be sterilized by boiling. On the perception of loss of virginity after usage of a menstrual cup, most of the participants (83.5%) responded that using a menstrual cup did not cause loss of virginity. Only four (3.7%) knew it could not be used during the postpartum period and 49 (45%) knew it could be used along with an IUD.

Concerns of the participants regarding the usage of menstrual cups are shown in Table 1.

**Table 1:** Concerns of the participants regarding the use of menstrual cups (n=109)

Concerns of participants about the usage of menstrual cups	Frequency (%)
Allergies/infection	17 (15.50%)
Difficulty in usage compared to other products	38 (34.80%)
Fear of insertion of a foreign body	64 (58.70%)
Difficulty in accessing the cups	9 (8.20%)
Discomfort	32 (29.30%)
Fear of losing virginity	4 (3.60%)
Limited knowledge	30 (27.50%)
Leakage	15 (13.70%)
Others	10 (9.10%)

Among the several concerns expressed by the participants, 58.7% were concerned about the insertion of a foreign material into their bodies.

The attitude of the participants towards the menstrual cup is shown in Table 2.

**Table 2:** Attitude of participants towards the menstrual cup (n=109)

Variables	Responses	Frequency (%)
I have tried to know more about the menstrual cup	Yes	82 (75.2)
	No	27 (24.8)
Menstrual cup is a better alternative to other sanitary products	Yes	100 (91.7)
	No	3 (2.8)
	I don’t know	6 (5.5%)
I would recommend the usage of menstrual cups to others	Yes	76 (69.7)
	No	5 (4.6)
	I don’t know	28 (25.7)
Likely to consider using a menstrual cup in the near future	Currently using it/used it	20 (18.4)
	Very likely	0
	Not at all likely	12 (11)
	I don’t know	77 (70.6)

The majority of the participants had a positive attitude towards the usage of the menstrual cup with 91.74% considering it a better alternative to other sanitary products but, 70.6% were unsure of whether they would consider using the menstrual cup in the near future.

Experiences of using the menstrual cup

Out of the 109 participants, only 20 (18.4%) had ever used the menstrual cup. Among them, 14 (12.8%) were continuing to use it. Table 3 shows the reasons for discontinuing the use of the cup.

**Table 3:** Reasons for discontinuing use of menstrual cups (n=6)

Reason for discontinuing usage of menstrual cup	Frequency (%)
Difficulty in insertion and removal	4 (66.66%)
		Difficulty in cleaning, insertion and removal	1 (16.67%)
				Difficulty in insertion, removal and pain	1 (16.67%)

The major reason for discontinuing the usage of a menstrual cup was difficulties in insertion and removal with all the participants who discontinued usage citing it.

The experiences of the participants who are continuing the usage of menstrual cups are shown in Table 4.

**Table 4:** Experience of current users of menstrual cups (n=14)

Variables	Response	Frequency(%)
Duration of usage of menstrual cup	< 6 months	6 (42.9)
	6- 12 months	5 (35.7)
	>1 year	3 (21.4)
Do you regularly use it in every cycle?	Yes	11 (78.6)
	No	3 (21.4)
Usage of other sanitary products along with menstrual cup on days with heavy flow	Yes	7 (50)
	No	7 (50)
More comfortable doing daily activities while using a cup	Yes	13 (92.86)
	No	1 (7.14)
Ease of using a cup	Very easy	7 (50)
	Easy after using a few times	6 (42.9)
	Difficult	1 (7.1)
Reasons for choosing menstrual cup over other products	Economical	13 (92.8%)
	Environment friendly	12 (85.7%)
	Increased comfort in doing daily activities	12 (85.7%)
	Less frequency in changing/cleaning	12 (85.7%)
	Less leakage	10 (71.4%)
	Others	1 (7.1%)
Methods used by current users for disposal of sanitary pads before usage of cup	Burning	9(64.29%)
	Disposal in public bins	1(7.14%)
	Flushing	3 (21.43%)
	Incineration	1(7.14%)
Likelihood of continuing usage of a menstrual cup	I will try and see for a few more cycles	2(14.3%)
	Very likely	12 (85.7%)
	Not likely	0

Around 50% of the users found it very easy to use the menstrual cup and 12 (85.7%) were very likely to continue using it. The cup's economical benefits, environmentally friendly properties, and increased comfort while doing daily activities were cited as major reasons by the users for choosing the menstrual cup.

## Discussion

This was a cross-sectional study conducted among female MBBS students of KMCT Medical College in Kozhikode, Kerala, India. A total of 109 students participated in the study, and the data was collected through an online questionnaire. The results showed that 12.8% of the participants were using menstrual cups, either alone or in conjunction with sanitary pads, while 93.6% were using sanitary pads on at least one day of their period and 80.7% were using only sanitary pads. Though the menstrual cup is an economical and eco-friendly alternative to sanitary napkins it has not yet gained much popularity even after online shopping facilities have made it increasingly accessible.

Most of the participants had good knowledge regarding the material of the cup, the frequency of emptying the cup, and the method of cleaning the cup. All of them knew that the cup could be reused. There seemed to be a lack of knowledge regarding the usage of cup during the post-partum period and with an intra-uterine device with more than half of the participants responding that they did not know about it. Though the study reflected a positive attitude among the participants towards the cup, there were several concerns regarding its use like fear of insertion. Difficulties in insertion and removal were cited as the most common cause for discontinuing the usage of the cup.

In a similar study conducted among medical students by Eti et al., none of the participants were using the menstrual cup. Around 45.96% were aware of the appropriate frequency of clearing the cup and only 28.4% chose boiling the cup as the measure for sterilizing it [13]. In another study conducted in Karnataka among women of reproductive age group only 80% of the participants were aware of the menstrual cup whereas all the participants in the present study were aware of it. Only 35.8% were aware of the frequency of clearing the cup as against 74.3% in the present study. Only 7.5% were aware of the appropriate method of cleaning the cup whereas 68.8% were aware in the present study [14].

In another study by Ballal et al. among women of reproductive age group, 2.6% were using the menstrual cup as against 12.8% in the present study. The main concerns expressed by the participants were the cumbersomeness of usage, poor availability, and cost [12]. In this study, the main concerns expressed were fear of insertion of the cup and difficulty in usage.

Various studies have shown that the usage of menstrual cups becomes easier in subsequent cycles. In a study conducted in Patna, the number of users reporting comfort of usage increased from 7.8% to 70.5% by the third cycle [15]. Another study conducted in Kerala also showed similar results with an increasing number of participants finding it easy to use the menstrual cup on repeated usage [16]. In a meta-analysis including 43 studies, it was found that in all qualitative studies, the adoption of menstrual cups required familiarisation over a few cycles and that peer support improved uptake [10]. In the present study also the participants expressed that the usage of the cup becomes easier after a few cycles.

In the present study, most of the participants were reluctant to use the cup due to concerns like fear of insertion, difficulty in usage compared to products like sanitary pads, and perceived discomfort, though most of them agreed that the cup is a better alternative to sanitary pads. Addressing these apprehensions through counselling and involving the menstrual cup users as peer educators can help in increasing the acceptance of the cup and sustaining its usage in the future.

## Conclusions

The study aimed to assess the knowledge, attitudes, and experiences of female medical students regarding menstrual cups. In total, 109 students participated in the study and most of them had considerable knowledge about the cup. Though most of them displayed a positive attitude towards the cup with the majority considering it a better alternative to other sanitary products, only a few of the participants were actually using it. The study revealed that there are several apprehensions regarding the cup that need to be addressed before it gains popularity. The study also highlights the need for education and promotion of menstrual cups as a better alternative to disposable menstrual products. 
